# Inhalation of the BK_Ca_-Opener NS1619 Attenuates Right Ventricular Pressure and Improves Oxygenation in the Rat Monocrotaline Model of Pulmonary Hypertension

**DOI:** 10.1371/journal.pone.0086636

**Published:** 2014-01-31

**Authors:** Marc Revermann, Skevi Neofitidou, Thomas Kirschning, Manuel Schloss, Ralf P. Brandes, Christian Hofstetter

**Affiliations:** 1 Department of Anesthesiology and Critical Care Medicine, University Hospital Mannheim, Faculty of Medicine, University of Heidelberg, Heidelberg, Germany; 2 Institute for Cardiovascular Physiology, Medical Faculty of the Goethe-University Frankfurt, Frankfurt, Germany; University of Illinois at Chicago, United States of America

## Abstract

**Background:**

Right heart failure is a fatal consequence of chronic pulmonary hypertension (PH). The development of PH is characterized by increased proliferation of vascular cells, in particular pulmonary artery smooth muscle cells (PASMCs) and pulmonary artery endothelial cells. In the course of PH, an escalated right ventricular (RV) afterload occurs, which leads to increased perioperative morbidity and mortality. BK_Ca_ channels are ubiquitously expressed in vascular smooth muscle cells and their opening induces cell membrane hyperpolarization followed by vasodilation. Moreover, BK activation induces anti-proliferative effects in a multitude of cell types. On this basis, we hypothesized that treatment with the nebulized BK channel opener NS1619 might be a therapy option for pulmonary hypertension and tested this in rats.

**Methods:**

(1) Rats received monocrotaline injection for PH induction. Twenty-four days later, rats were anesthetized and NS1619 or the solvent was administered by inhalation. Systemic hemodynamic parameters, RV hemodynamic parameters, and blood gas analyses were measured before as well as 30 and 120 minutes after inhalation. (2) Rat PASMCs were stimulated with PDGF-BB in the presence and absence of NS1619. AKT, ERK1 and ERK2 activation were investigated by western blot analyses, and relative cell number was determined 48 hours after stimulation.

**Results:**

Inhalation of a 12 µM and 100 µM NS1619 solution significantly reduced RV pressure without affecting systemic arterial pressure. Blood gas analyses demonstrated significantly reduced carbon dioxide and improved oxygenation in NS1619-treated animals pointing towards a considerable pulmonary shunt-reducing effect. In PASMC’s, NS1619 (100 µM) significantly attenuated PASMC proliferation by a pathway independent of AKT and ERK1/2 activation.

**Conclusion:**

NS1619 inhalation reduces RV pressure and improves oxygen supply and its application inhibits PASMC proliferation *in vitro*. Hence, BK opening might be a novel option for the treatment of pulmonary hypertension.

## Introduction

Pulmonary hypertension (PH) is a serious disease with a fatal progressive course and a risk of perioperative morbidity and mortality. Although pharmacological treatment improved in recent years, the survival of patients suffering from PH is limited by right heart failure and arrhythmias [1]. Current PH treatment strategies rely on vasodilatory and anti-proliferative agents, e.g. prostaglandin-analogues, phosphodiesterase type 5 inhibitors and endothelin-receptor antagonists [2]. Since drugs which exclusively dilate the pulmonary vasculature are not available yet, inhalative application of vasoactive compounds is a promising approach to bring on selective pulmonary vasodilation with few systemic side effects. However, although some inhalatively applicable drugs have been developed, PH is still a therapeutic challenge with problematic prognosis. Thus, new therapeutic strategies to improve the survival of patients suffering from this disease are still required.

An ideal drug for PH therapy has to fulfil the following two requirements: 1. Improvement of right ventricular performance. 2. Checking the disease’s long-term progression by limiting the uncontrolled pulmonary arterial vascular cell proliferation, particularly pulmonary artery smooth muscle cell (PASMC) proliferation.

Right ventricular dysfunction and failure are the limiting factors concerning the prognosis of pulmonary hypertension. Right ventricular (RV) afterload, contractility and relaxation are parameters, which characterize right ventricular function. Here, determination of appropriate parameters, such as RV pressure, dP/dt _max_, and dP/dt _min_ help to assess RV function.

In this regard, the velocity of pressure rise (dP/dt_max_) is a parameter which is dependent on right ventricular contractility and right ventricular afterload, whereas dP/dt_min_ is a parameter which is useful for determination of ventricular relaxation.

Moreover, right ventricular oxygen demand-supply-ratio is an objective of PH treatment. Concerning the latter, right ventricular myocardial oxygen demand depends on right ventricular work, which in turn mainly depends on the right ventricular afterload. Additionally, PH is usually characterized by an increased pulmonary shunt volume, which results in a reduced oxygenation state and general hypoxemia.

Potassium ion channels are involved in numerous cardiovascular functions. In vascular cells their opening leads to hyperpolarization of the cell membrane and induces a multitude of changes in the cellular activity. In endothelial cells hyperpolarization increases calcium influx and enhances endothelial NO synthase activation [3], and in smooth muscle cells hyperpolarization induces relaxation by reducing the open probability of L-type calcium channels [4]. This in turn provokes vasodilation and decreases the activity of – at least partly – pro-proliferative calcium dependent mitogen activated protein (MAP) kinases.

The BK channel is a calcium-sensitive potassium ion channel, which is ubiquitously expressed in blood vessels, especially in endothelial and vascular smooth muscle cells [3], [5]. Among the family of calcium-dependent potassium channels, BK channels show the most substantial potassium efflux. Since potassium channels modulate the vascular tone [6] and the cellular proliferative state [7], we investigated the impact of the BK channel opener NS1619 on right ventricular and systemic hemodynamic performance and blood gases in the rat monocrotaline PH model as well as on PASMC proliferation in cell culture.

## Materials and Methods

### Animals and Study Protocol

Animal experiments were performed in accordance with the National Institutes of Health Guidelines on the Use of Laboratory Animals. Both the University Animal Care Committee and the Federal Authorities for Animal Research of the Regierungspräsidium Darmstadt (Hessen, Germany) approved the study protocol. Male Sprague-Dawley rats (300–350 g body weight) were obtained from Charles River Laboratories (Sulzfeld, Germany). Rats were exposed to Monocrotaline (Sigma, Deishofen, Germany), which was dissolved in HCl (0.1 mol/l), adjusted to pH 7.4 with NaOH solution (0.1 mol/l) and administered as a single subcutaneous injection (60 mg/kg body weight) as described previously [8]. After twenty-four days, rats (440,8±33,72 g body weight) were randomized to the different study groups. After anesthesia with pentobarbital (Narcoren, Merial, Halbergmoos, Germany; 50 mg/kg, i.p.) plus fentanyl (Janssen-Cilag, Neuss, Germany; 0.05 mg/kg, i.p.), rats were weighed and then placed supine on a heating pad. A tracheotomy was performed, and a 14-gauge cannula (inner diameter 2.0 mm, outer diameter 2.5 mm, Abbott, Wiesbaden, Germany) was inserted to ventilate the animals with an infant ventilator (Stephanie, Stephan, Gackenbach, Germany). The following parameters were used in a pressure-controlled ventilation protocol: initial maximum pressure 16 cm H2O; positive endexpiratory pressure 4 cm H2O; respiratory rate 30 breaths/min; time inspiratory/expiratory: 1/1; FiO2 0.21. Body temperature was monitored by a rectal probe and kept constant at 37.0–38.0°C throughout the experiment. Fluid-filled polyurethane catheters (inner diameter 0.58 mm, outer diameter 0.96 mm, SIMS Portex, Hythe, UK) were inserted in the right femoral vein for infusion of anesthetics and in the right femoral artery for invasive blood pressure measurement and withdrawal of blood samples, respectively. After anesthetic dosage titration, anesthesia was maintained by continuous intravenous infusion of pentobarbital (5–10 mg/kg/hr) and fentanyl (2.5–5 µg/kg/hr). With the help of a skin incision the right jugular vein was exposed and a catheter (Millar, 2F; ADInstruments, Spechbach, Germany) was inserted into the vessel and advanced to the right ventricle. Inspiratory pressure levels were adjusted to achieve standardized basal carbon dioxide values (34–46 mmHg). Afterwards, no further changes of the lung ventilator settings were performed. Systemic arterial and right ventricular pressure curves and their derivatives, and body temperature were recorded permanently using a data acquirement system (Powerlab; ADInstruments, Germany). Volume reagibility parameters were calculated as follows: Systolic blood pressure variation (SPV) = bp_sys_max−bp_sys_min/bp_sys_mean, pulse pressure variation (PPV) = PPmax−PPmin/PPmean, relative stroke volume variation (SVV) = RR_integral_ max – RR_integral_ min/RR _integral_ mean. Following the principles of pulse contour analysis we determined the changes of the integral below the arterial pressure curve and describe the changes of that area as “relative stroke volume” (rSV) or as “relative stroke volume variation” (rSVV).

Thirty-one rats were randomized. Four animals were excluded because of technical, respectively experimental problems (e.g. catheter dislocation). Only rats with overt pulmonary hypertension (RVSP values 35–75 mmHg) were included in the study. Three of the remaining twenty-seven animals were excluded because their RVSP was not within the target range. The RVSP of healthy male Sprague Dawley rats is approximately 25 mmHg [9]. Data were continuously recorded and statistical analysis was performed for data average of a 60 seconds period once hemodynamic steady state was reached for 3 minutes. Blood gas analyses of animals with initial normocapnia (PaCO_2_ 34–46 mmHg) were recorded (Radiometer ABL 80 flex, Radiometer GmbH, Willich, Deutschland) before, 30 and 120 minutes after compound nebulization and statistical analysed.

### Nebulization

For the nebulization, NS1619 (Sigma, Deishofen, Germany) was dissolved in Dimethyl Sulfoxide (DMSO; Sigma, Deishofen, Germany) and expanded with sodium chloride solution, resulting in 3 ml of 100 µM (100 µM group) and 12.5 µM (12 µM group) NS1619 solutions. These solutions were applied by an integrated nebulizer (multisonic infracontrol, Schill, Probstzella, Germany) which produced particles with a median size of 2.0 µm and an intrapulmonary deposition fraction of 3.8% (manufacturer information) for a three-minute period.

### 3-(4,5-Dimethylthiazol-2-yl)-2,5-diphenyltetrazolium Bromide Pulmonary Arterial Smooth Muscle Cells Proliferation Assay

Rat pulmonary artery smooth muscle cells (PASMCs) were seeded in multiwell plates in cell culture medium (8%FCS) after isolating them from rat pulmonary arteries as described previously [8]. When a subconfluent growth state was reached, culture medium was replaced by a 8% FCS containing medium, and NS1619 (100 µmol/l final concentration; Sigma-Aldrich, Germany) or the solvent (DMSO) were added to subgroups. Their proliferation was subsequently stimulated by platelet-derived growth factor-BB (PDGF-BB; 20–30 ng/ml). After two days, the cell number was determined by the MTT test. In brief, the plates were washed and (3-(4,5-dimethylthiazol-2-yl)-2,5-diphenyltetrazolium bromide (MTT, Sigma) was added. After an incubation period of two hours (37°C, 5% CO_2_), acidified isopropanol was added to dissolve the precipitated formazan. Absorbance was determined by a spectrophotometer (550 nm wavelength; Wallac Victor; EG&G Wallac, Freiburg, Germany).

### Immunoblotting

For the western blot analysis rat PASMCs were isolated from rat pulmonary arteries and seeded in 3.5 cm dishes until they reached a nearly confluent growth state. PASMCs were starved for 24 hours (0.1% BSA) and afterwards stimulated with PDGF-BB (10 ng/ml) in the absence or presence of NS1619 (100 µmol/l; 120 minutes preincubation, Sigma-Aldrich, Germany). After four and eight minutes, respectively, the cells were frozen in liquid nitrogen and subsequently scraped in triton-X-100 lysis buffer. Protein concentration was determined by Bradford assay. Extracted protein samples were boiled in a Laemmli buffer and 50 µg of each sample was separated by sodium dodecyl sulfate–polyacrylamide gel electrophoresis (SDS–PAGE; 10%) and transferred onto nitrocellulose membrane as described previously [8]. Proteins were detected using primary antibodies against p-ERK1, p-ERK2, ERK1, ERK2 and p-AKT (Cell Signaling, Danvers, USA;1∶1.000 each antibody) and appropriate secondary antibodies labeled with infrared dyes and visualized using the Odyssey infrared imaging system (Li-COR Biosciences, Bad Homburg, Germany). Densitometry was carried out using the integrated Odyssey software.

### Data and Statistical Analysis

*S*tatistical analyses were performed by using Kolmogorov-Smirnov normality test and analysis of variance (ANOVA), followed by Tukey-corrected Fisher’s LSD test and the unpaired t-test for normal distributed data and Kruskal-Wallis test with Dunn's post test or, where appropriate, the Mann-Whitney test, for nonparametric distributed data. A P value of less than 0.05 was considered statistically significant.

## Results

### Inhalation of NS1619

In this study, we explored whether the BK channel opener NS1619 could affect hemodynamics with focussing on the right ventricle as its failure is the life-threatening consequence of PH. To minimize systemic side effects and optimize the drug’s concentration in the target organ, NS1619 or the solvent were applied by inhalation. As the current study investigates both, a possible biologic effect of inhaled NS1619 on cardiopulmonary function in principle, and a possible dose dependency, we performed two separate statistical analyses: 1. Dose-dependent effects were examined by comparing all three study groups. 2. General NS1619-dependent effects were examined by comparing all NS1619-treated animals (∑NS1619 = ∑) with the control group.

The basic values for each of the parameter recorded did not differ significantly between the three groups (Solvent, 12 µM NS1619, 100 µM NS1619) **(** **Table 1** **)**.

**Table 1:** Basic values.

Parameter	Solvent	12 µM NS1619	100 µM NS1619
**RVSP (mmHg)**	51,59±3,868	48,54±2,388	51,78±2,958
**RVMP (mmHg)**	22,65±1,697	23,06±1,088	23,66±2,148
**RVDP (mmHg)**	2,466±1,200	4,143±1,322	2,629±1,504
Heart rate * RVSP	19863±1439	18514±1621	17875±1109
dP/dt _max_ (mmHg * s^−1^)	1913±122,7	2043±251,6	1619±162,8
dP/dt _min_ (mmHg * s^−1^)	−1824±115,8	−1723±162,2	−1523±139,1
**heart rate (bpm)**	387,3±14,73	378,7±22,35	345,8±9,934
**bp sys (mmHg)**	149,4±5,292	148,7±5,031	163,3±10,76
**bp mean (mmHg)**	111,9±4,868	108,6±5,447	131,0±9,340
**bp dia (mmHg)**	98,78±4,670	85,66±5,017	110,8±7,863
**rSVV**	0,1712±0,01687	0,2048±0,04766	0,1667±0,02251
**SPV**	0,1594±0,01857	0,1602±0,03862	0,09328±0,01192
rSV (mmHg* s)	16,88±1,051	17,28±1,902	19,86±3,363
**PPV**	0,2026±0,02420	0,1761±0,04488	0,1622±0,02647
Heart rate * rSV (min^−1^ *mmHg*s)	6446±289,1	6313±328,0	6757±1039
Heart rate * RR sys (min^−1^ * mmHg)	57947±3102	55948±2694	56143±3292
**dP/dt _max_ (mmHg xs^−1^)**	3367±286,4	3430±353,9	3312±346,2
**PaO_2_ (mmHg)**	80,73±3,136	69,27±2,089	72,83±4,362
**PaCO_2_ (mmHg)**	40,39±1,481	41,17±1,486	40,67±1,430
**HCO_3_^−^ (mmol/l)**	24,84±0,5464	24,48±0,8220	25,63±1,119
**Base excess (mmol/l)**	0,6000±0,6541	0,06667±0,6839	1,117±1,364
**pH**	7,408±0,01601	7,392±0,00654	7,412±0,02535

The basic values for each of the parameter recorded did not differ significantly between the three groups (Solvent, 12 µM NS1619, 100 µM NS1619).

### Inhalation of NS1619 Reduces Right Ventricular Afterload

**Figure 1:**
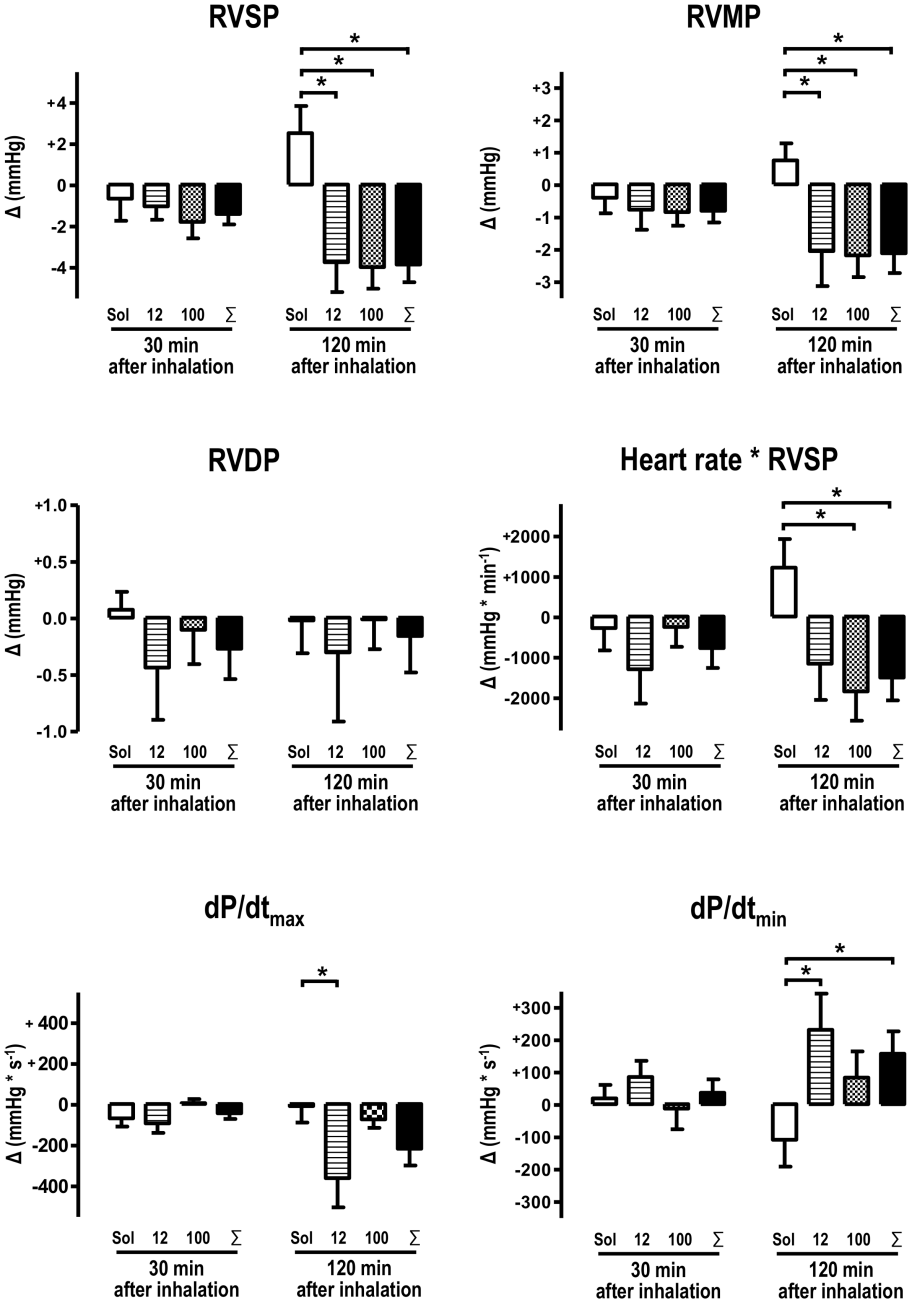
NS1619 reduces right ventricular pressure. Inhalation of 12 µM and 100 µM NS1619 significantly attenuates monocrotaline-induced right ventricular hypertension in comparison to solvent-treated animals 120 minutes after inhalation. In detail, right ventricular systolic pressure (RVSP) and right ventricular mean pressure (RVMP) were reduced in both NS1619 groups and consecutively in the cumulative ∑NS1619 versus solvent calculation. As a result of the reduced RVSP, the approximated right ventricular work, which is characterized by the product of heart rate and RVSP is attenuated in the 100 µM and the ∑NS1619 group, too. Right ventricular maximum velocity of the pressure slope (dP/dt_max_) were significantly attenuated in the 12 µM group 120 minutes after inhalation. Moreover, 12 µM NS1619 as well as the ∑NS1619 group exhibit a significant diminishment of the right ventricular relaxation velocity by an increase of the dP/dt_min_-slope. Data obtained after inhalation were subtracted from individual basis values and individual value differences (Δ) were calculated for each animal and time point. RVDP: right ventricular diastolic pressure, dP/dt_max_: maximum contraction velocity (Solvent: n = 10; 12 µM: n = 7, 100 µM: n = 7; ∑NS1619: n = 14;*: p<0.05).

Twenty-four days after the monocrotaline challenge, PH was established. This was shown by an elevated average right ventricular systolic pressure (RVSP) of 50,8 mmHg and an average right ventricular mean pressure (RVMP) of 23 mmHg **(** **Table 1** **)**. 120 minutes after inhalation, the NS1619 treatment reduced right ventricular pressure significantly **(** **Figure 1** **)**. Accordingly, right ventricular myocardial work, defined by the product of heart rate and right ventricular afterload (RVSP), decreased in animals treated with 100 µM NS1619 and in the ∑NS1619 group. Right ventricular maximum velocity of the pressure slope (dP/dt_max_) were significantly attenuated in the 12 µM group 120 minutes after inhalation. The maximum velocity of the pressure drop (dP/dt_min_), on the other hand, increased after inhalation of NS1619 and reached statistical significance in the 12 µM group as well as in the pooled NS1619 group compared to control animals **(** **Figure 1** **)**.

### Inhalation of NS1619 does not Affect Systemic Hemodynamic Parameters

30 and 120 minutes after inhalation, the results for heart rate, arterial systolic, mean and diastolic blood pressure did not differ between subjects receiving NS1619 or subjects receiving the solvent **(** **Table 2** **)**.

**Table 2:** Systemic hemodynamic parameters.

	Solv.	12 µM	100 µM	∑ NS1619	Solv. vs.∑ NS1619	Solvent	12 µM	100 µM	∑ NS1619	Solv. vs.∑ NS1619
**heart rate (bpm)**	−0,9±8,6	−19,2±17,3	+6,9±6,7	−6,2±9,6	P = 0,70	+4,2±6,7	+5,6±11,1	−9,5±13,7	−1,9±8,7	P = 0,61
**bp sys (mmHg)**	−2,2±5,1	−4,7±5,9	+0,9±5,6	−1,9±4,0	P = 0,96	−0,0±10,0	−5,1±7,1	−1,7±9,1	−3,4±5,6	P = 0,75
**bp mean (mmHg)**	+0,6±2,7	+0,0±4,0	+3,2±4,1	+1,6±2,8	P = 0,79	+2,3±8,8	−0,4±6,6	+1,0±9,2	+0,3±5,5	P = 0,84
**bp dia (mmHg)**	+0,7±2,7	+0,7±3,6	+3,7±3,2	+2,2±2,4	P = 0,68	+2,0±8,9	+2,0±6,3	+2,2±7,9	+2,1±4,8	P = 0,99
	**(Δ) 30 min after inhalation**					**(Δ) 120 min after inhalation**				

Heart rate, systolic (bp sys), mean (bp mean), diastolic (bp dia) arterial blood pressure were determined before (basis) as well as 30 and 120 minutes after inhalation of two different NS1619 concentrations, respectively solvent. Data obtained after inhalation were subtracted from individual basis values and individual value differences (Δ) were calculated for each animal and time point. None of the parameters investigated were affected by NS1619 inhalation in a significant manner. According to the character of the study as “a proof of principle”, pooled NS1619 (∑NS1619) data was compared with the control group (Solvent = Solv.). No NS1619-dependent effect on systemic hemodynamic parameters was discovered.

### Inhalation of NS1619 does not Affect Predictors of Fluid Responsiveness

To explore possible alterations of fluid responsiveness predictors, which are more difficult to determine than systemic arterial blood pressure, we determined the changes of relative stroke volume (rSV), relative stroke volume variation (rSVV), systolic pressure variation (SPV) and pulse pressure variation (PPV). Concerning these parameters, there was no significant difference between NS1619 treated and control animals **(** **Table 3** **)**.

**Table 3:** Intravascular volume parameters.

	Solvent	12 µM	100 µM	∑ NS1619	Solv. vs.∑ NS1619	Solvent	12 µM	100 µM	∑ NS1619	Solv. vs.∑ NS1619
**rSVV**	+0,00±0,01	−0,03±0,04	−0,01±0,02	−0,02±0,02	P = 0,44	+0,02±0,02	+0,01±0,03	−0,04±0,02	+0,02±0,02	P = 0,18
**SPV**	−0,01±0,02	+0,03±0,04	+0,01±0,01	+0,02±0,02	P = 0,31	+0,02±0,02	+0,05±0,03	+0,01±0,01	+0,03±0,02	P = 0,76
rSV (mmHg* s)	+0,04±0,52	+2,93±3,02	−0,22±0,74	+1,35±1,56	P = 0,50	+0,49±1,20	−0,58±0,97	+0,28±1,42	−0,15±0,84	P = 0,66
**PPV**	+0,01±0,01	+0,08±0,08	−0,01±0,01	+0,04±0,04	P = 0,38	+0,03±0,03	+0,09±0,04	−0,00±0,02	+0,05±0,03	P = 0,72
	**(Δ) 30 min after inhalation**					**(Δ) 120 min after inhalation**				

For the investigation of fluid responsive parameters were calculated: relative stroke volume (rSV) characterized by the area under the arterial blood pressure curve, systolic pressure variation (SPV), relative stroke volume variation (rSVV), and pulse pressure variation (PPV). No NS1619-dependent effect on these parameters was discovered. Data obtained after inhalation were subtracted from individual basis values and individual value differences (Δ) were calculated for each animal and time point.

### Inhalation of NS1619 does not Affect Left Ventricular Hemodynamic Parameters

For estimating left ventricular work, the product of heart rate and systolic arterial blood pressure was calculated. Here, no significant difference between the three groups was found. Furthermore, the analysis of the blood pressure curve’s dP/dt _max_, which depends on left ventricular contractility and vascular compliance, did not show significant variation after NS1619 inhalation. To assess the cardiac output the product of heart rate and the integral below the arterial blood pressure curve was calculated. No compound-dependent alteration was found **(** **Table 4** **)**.

**Table 4:** Left ventricular work.

	Solvent	12 µM	100 µM	∑ NS1619	Solv. vs.∑ NS1619	Solvent	12 µM	100 µM	∑ NS1619	Solv. vs.∑ NS1619
Heart rate *		+643±	+140±						+8±	
**rSV**	+29±154	668	231	+392±345	P = 0,41	+276±481	−8±386	+25±532	316	P = 0,63
(min^−1^ *mmHg*s)										
Heart rate *							–	–		
**RR sys**	−966±2433	−4232±	+1719±	−1257±2395	P = 0,93	+452±406	1100±3788	1299±4684	−1199±	P = 0,74
(min^−1^ * mmHg)		3948	2513						2894	
**dP/dt _max_**	−201±377	−570±	−157±	−364±235	P = 0,70	−633±457	−454±451	−548±444	−501±	P = 0,80
**(mmHg xs^−1^)**		341	329						304	
	**(Δ) 30 min after inhalation**					**(Δ) 120 min after inhalation**				

Left ventricular work was estimated as the product of heart rate (HR) and systolic arterial pressure (bp sys). Relative cardiac output was estimated as the product of heart rate and relative stroke volume (integral of the arterial curve). dP/dt _max_ of the arterial pressure curve did not show a NS1619-dependent variation. Data obtained after inhalation were subtracted from individual basis values and individual value differences (Δ) were calculated for each animal and time point. (Solvent: n = 10; 12 µM: n = 7, 100 µM: n = 7; ∑NS1619: n = 14;*: p<0.05).

### NS1619 Inhalation Reduces Dissolved Carbon Dioxide and Increases Dissolved Oxygen in the Blood

**Figure 2:**
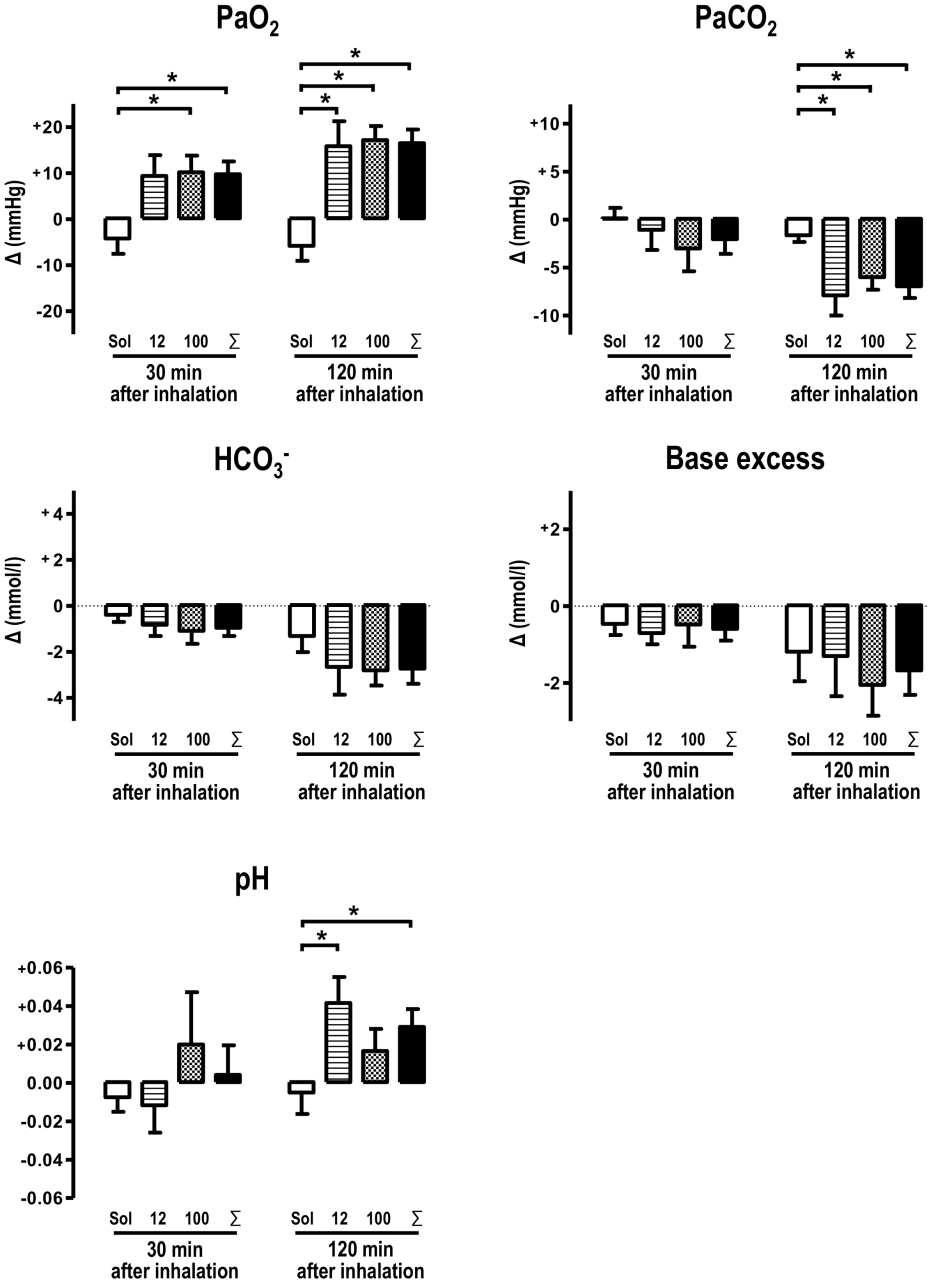
Blood gas analyses. 12 µM and 100 µM NS1619 improved oxygenation (PaO_2_) considerably 120 minutes after inhalation and 100 µM and ∑NS1619 data exhibits a significant increase already 30 minutes after NS1619 inhalation. Carbon dioxide levels (PaCO_2_) were attenuated 120 minutes after NS1619 inhalation. 12 µM and ∑NS1619 data demonstrate an elevated pH 120 minutes after inhalation. Base excess (BE) and bicarbonate concentration were not altered significantly by NS1619 although HCO_3_^−^ was attenuated in NS1619 treated animals at least by trend. Data obtained after inhalation were subtracted from individual basis values and individual value differences (Δ) were calculated for each animal and time point. (Solvent: n = 8; 12 µM: n = 6, 100 µM: n = 6, ∑NS1619: n = 12; *: p<0.05).

For evaluating the impact of BK channel opener on gas exchange and oxygen supply, blood gas analyses were performed before as well as 30 and 120 minutes after inhalation. Initial values of blood gas analyses did not differ between the three groups investigated. 120 minutes after inhalation of NS1619, carbon dioxide levels were significantly reduced in animals which were treated with NS1619 **(** **Figure 2** **)**. On the other hand, the oxygen level was considerably increased 30 and 120 minutes after NS1619 inhalation. This effect reached high significance level after 120 minutes **(** **Figure 2** **;** p<0.05). Also, 120 minutes after inhalation, pH was significantly increased in the pooled NS1619 group, indicating the development of a respiratory alkalosis. Base excess and bicarbonate values did not differ significantly between the three groups.

### The BK_Ca_ Channel Opener NS1619 Reduces Pulmonary Artery Smooth Muscle Cell (PASMC) Proliferation

**Figure 3:**
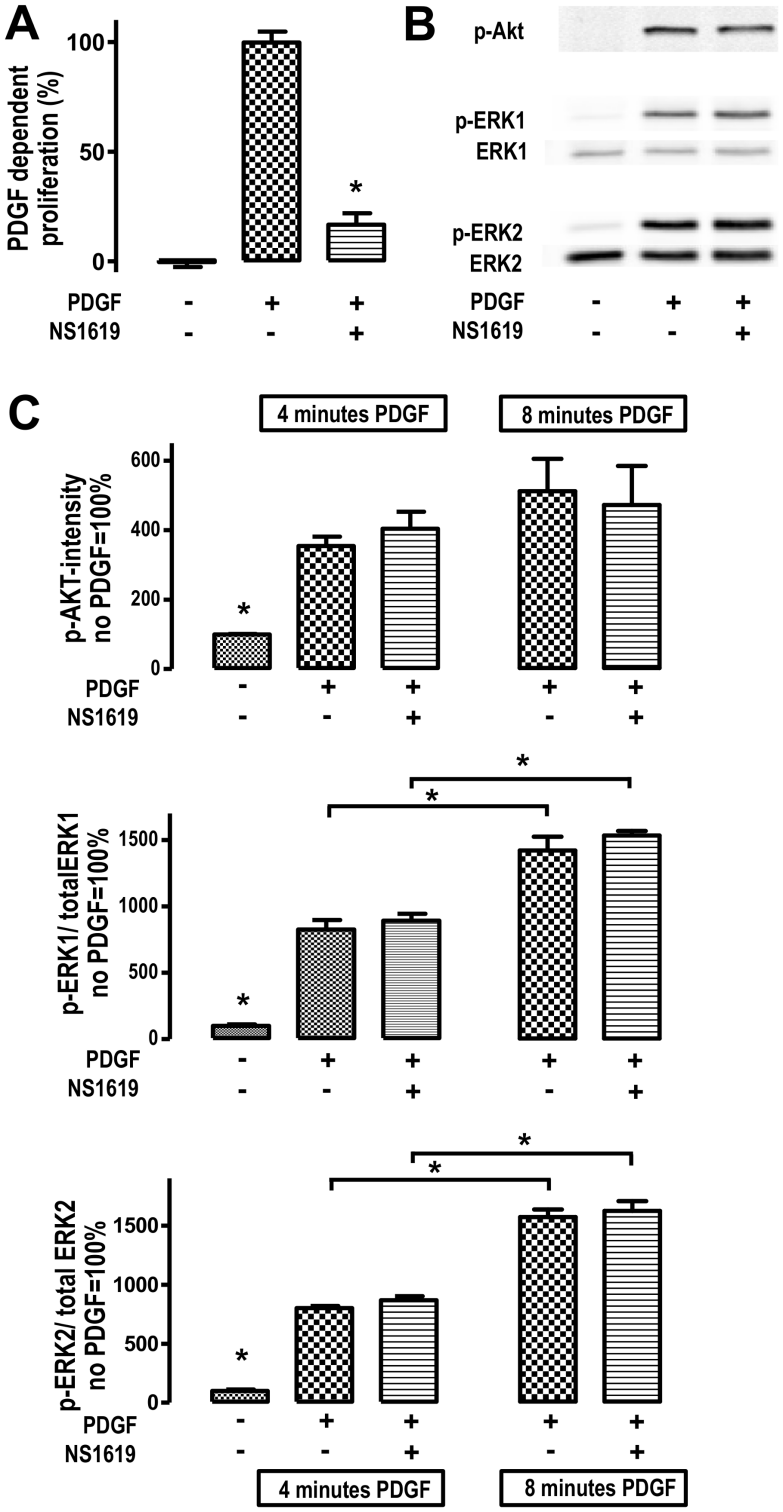
NS1619 attenuates PDGF-induced PASMC proliferation. (A) BK channel opening agent NS1619 (100 µmol/l) reduces PDGF-induced PASMC proliferation (n = 21 each column; * p<0.05). (B,C) In cultured PASMCs, phosphorylation state of protein kinases ERK1, ERK2 and AKT increased significantly 4 minutes after PDGF-BB stimulation (n = 3–6, *:p<0.05). Afterwards, phosphorylation states of ERK1 and ERK2 significantly increased within the period between 4 and 8 minutes after PDGF stimulation (n = 3–6; *:p<0.05) whereas AKT phosphorylation state rose by trend. BK channel opening by NS1619 (100 µmol/l) treatment does not affect phosphorylation of any kinase analysed.

To test the hypothesis that BK channel opening affects PASMC proliferation, we investigated the effect of NS1619 on PDGF-induced PASMC proliferation *in vitro* **(** **Figure 3** **)**. The platelet-derived growth factor (PDGF-BB) was applied as stimulus as it is a well-known contributor to formation of some PH subspecies, in particular development of pulmonary arterial hypertension (PAH). In fact, 100 µM NS1619 attenuated PDGF-dependent PASMC proliferation significantly **(** **Figure 3A** **)**.

### The Antiproliferative Effect of NS1619 is Independent of MAP Kinases

Since mitogen-activated protein kinases ERK1, ERK2 and AKT are important elements of PDGF’s intracellular signaling pathway, we performed western blot analyses to find out whether those kinases mediate the anti-proliferative effect of NS1619. Accordingly, in cultured PASMC’s, activation of ERK1, ERK2 and AKT was investigated by measuring the phosphorylation state four and eight minutes after the PDGF-BB challenge **(** **Figure 3B** **)**. Phosphorylation of ERK1, ERK2 and AKT significantly increased four minutes after PDGF stimulation **(** **Figure 3C** **;** *: p<0.05). In the period between four and eight minutes after stimulation, ERK1 and ERK2 phosphorylation increased time-dependently (*:p<0.05) whereas AKT phosphorylation increased by trend within this period. NS1619 (100 µmol/l) did not affect ERK1, ERK2 and AKT activation state after PDGF-BB challenge **(** **Figure 3B,C** **)**.

## Discussion

The present study focused on the acute effect of nebulized BK channel opening agent NS1619 on the cardiopulmonary system, i.e. pharmacologically induced changes of right ventricular performance, systemic hemodynamic parameters, and blood gas exchange of rats with experimentally induced pulmonary hypertension. Additionally, we investigated the impact of the BK channel opening compound on pulmonary arterial smooth muscle cell proliferation *in vitro*.

NS1619 reduced right ventricular afterload without affecting systemic hemodynamic parameters. Blood gas analyses revealed an increase of dissolved oxygen and a decrease of dissolved carbon dioxide and development of a respiratory alkalosis after pharmacological BK opening.

Potassium channel generate the basis of the cellular resting membrane potential in numerous cell types and are involved in organization of the contractile tone of vascular smooth muscle cells [10]–[13]. Calcium-dependent BK_Ca_ channels occur ubiquitously in the vascular system and conduct ionic currents that mediate membrane hyperpolarization causing vasodilation [6]. They are regulated by calcium, but also by a number of other factors, such as cellular membrane potential and channel phosphorylation [6], and have recently been detected in pulmonary artery smooth muscle cells [14,] [15].

Since NS1619 is described as a vasodilating and anti-proliferative compound, we investigated its impact in the rat monocrotaline model of pulmonary hypertension. To do so, NS1619 was administered by inhalation as this application modality is preferable to systemic application concerning reduced systemic side effects and high local concentrations at the target site [16]. Accordingly, the failing right ventricle is very sensitive to low systemic arterial blood pressure.

In this context, systemic BK ion channel opening may attenuates the protective Euler-Liljestrand mechanism by causing an increased pulmonary shunt volume followed by impaired oxygenation.

In the present study inhalative application of NS1619 did not affect heart rate, systemic arterial pressure and volume state indicating parameters, like pulse pressure variation and systolic pressure variation.

Two hours after solvent inhalation, compared to the baseline measurements, right ventricular systolic pressure (RVSP) and right ventricular mean pressure (RVMP) were increased in control animals indicating a successive mismatch of ventilation-perfusion ratio in artificially ventilated animals.

The latter effect seemed to be compensated by NS1619 inhalation, which is characterized by a significant RVSP and RVMP reduction in NS1619-treated animals. The delayed achievement of a significance level could be explained by the long diffusion distance and the diminished relaxation potential of the multilayered vascular medial layer. Interestingly, right ventricular rate-pressure product is attenuated in NS1619-treated animals, presumably resulting in a reduced right ventricular oxygen demand.

Some well known pulmonary vascular dilators, like NO and prostaglandins, could activate BK channel activity via cGMP and cAMP dependent protein kinase phosphorylation [17] ^,^ [18]–[20]. Accordingly, the established PH therapy strategies are at least partially dependent on BK activation.

For this reason, direct activation of a primary pharmacological target in PH therapy was a goal of this study. Neither of the NS1619 concentrations chosen for the *in vivo* study part, produced significantly different results. This may be accounted for by a pharmacological ceiling effect, which apparently already occurred at 12 µM. Thus, an increase from 12 to 100 µM did not improve either hemodynamic performance or ventilation-perfusion-ratio.

Two hours after inhalation we observed a decrease of the right ventricular maximum velocity of the pressure slope (dP/dt_max_) in the 12 µM group. This effect could be caused by a decreased afterload, which has been reported previously and indicate a reduced right ventricular work in consequence of PH treatment [21]–[23]. Accordingly, we observed an increase of the relaxation velocity (dP/dt_min_), which frequently accompanies a treatment-induced dP/dt_max_ reduction [21]–[23]. The reduced right ventricular lusitropy, characterized by dP/dt_min_ increase, was found in the 12 µM group and in the pooled NS1619 group. To our knowledge, there is no immediate connection of BK channels and the three main lusitropy mediating cardiomyocytical enzyme systems (Calcium ATPase, Na-Ca exchanger and the sarcoplasmic reticulum calcium ATPase, SERCA) observed so far and, additionally, BK channels are supposed to be absent from the cardiac myocyte sarcolemmal membrane [24]. This indicates that afterload reduction influences right ventricular lusitropy, perhaps mediated by a diminished sympathoadrenergic drive following RV pressure relief. Furthermore, the demonstrated acute attenuation of right ventricular rate-pressure-product could be caused by a reduced adrenergic stimulation of SERCA leading to an increase of dP/dt_min_.

Two hours after inhalation, carbon dioxide plasma level was significantly reduced and corresponding oxygen plasma level was significantly increased in NS1619-treated animals. Besides a putative bronchodilatory effect of NS1619 [25] ^,^ [26], this phenomenon is possibly due to reduction of pulmonary arterio-venous shunt volume. Here, a disproportional oxygen increase over carbon dioxide decrease could be either caused by carbon dioxide replenishment from bicarbonate and/or a consequence of the reduced pulmonary shunt volume. Since we were not able to take mixed-venous blood samples, shunt calculation with the help of the Berggren formula could not be applied. However, we assume that the net oxygenation rise is due to pulmonary shunt reduction which may underlines the beneficial impact of NS1619 on PH disease.

Intracellular calcium augments smooth muscle cell contraction and stimulates cell proliferation [27] which could be mediated by calcium-dependent ras-activation resulting in phosphorylation and activation of mitogen activated protein kinases, such as ERK1 (p44), ERK2 (p42) and AKT [28], [29]. BK channel activation reduces intracellular calcium concentration via smooth muscle cell membrane hyperpolarization and consecutive inhibition of l-type calcium channels [30]. Pulmonary vascular remodeling is – at least partially - initiated or mediated by the platelet derived growth factor (PDGF) [31]–[34]. In this regard, PDGF antagonism is possibly a promising treatment strategy, especially for humans suffering from pulmonary arterial hypertension (PAH/group I, WHO PH classification) [34]. This in mind, we used PDGF as stimulus for pulmonary artery smooth muscle cell (PASMC) proliferation *in vitro*, which represents a cellular model for PAH initiation *in vivo* [35]. However, recently published data indicates that PDGF inhibition ameliorates exercise capacity and hemodynamics in PAH patients but without affecting mortality and functional class affiliation [36].

In the present study, 100 µmol/l NS1619 reduced PDGF-induced PASMC proliferation reliably and significantly. According to other studies, 30 to 100 µmol/l NS1619 generates a general anti-proliferative effect, which has been shown in diverse cellular types [37]–[39]. Also, recently published data concerning the effect of NS1619 on vascular smooth muscle cell proliferation underlines the impact of BK channel activation on vascular remodeling [39]. Since intracellular calcium content is reduced after BK channel-mediated hyperpolarization, our hypothesis was that the calcium dependent ras/ERK/AKT pathways perhaps convey the anti-proliferative effect and performed cell culture experiments without detecting an alteration of ERK1, ERK2 and AKT phosphorylation depending on NS1619 exposure. Concerning this matter, we can only speculate whether anti-proliferative properties of NS1619 are due to mitochondrial membrane effects. Here, it has been reported that the opening of mitochondrial potassium channels has a benefit in the context of ischemic and anesthetic cardiac preconditioning [40]–[42]. Moreover, potassium channel opening has been proposed for mediating smooth muscle cell proliferation reduction and apoptosis induction, which, in turn, could be an essential mechanism for reducing pulmonary vascular wall remodeling in PAH [43], [44].

### Limitations

- Initially we wanted to investigate the effect of NS1619 in two PH models, namely in the MCT model and in the thromboxane-analogue U46619-induced PH model. But although we applied U46619 doses up to 6000 ng/100 g/min, which was sixfold higher than described previously [45], we were not able to induce a robust right ventricular hypertension. Instead of that, we observed a massive bronchial obstruction and so we stopped this study section.
- Although the monocrotaline model of PH is well established, the results obtained in this animal model have to be considered carefully because of the different features of vascular remodeling in rats and humans. In humans, endothelium-derived vascular alterations, like plexiform lesions, are pathognomonic for pulmonary arterial hypertension whereas MCT-treated animals show mainly medial layer alterations [46]. Because of the problematic expression of BK channels in cultured endothelial cells [47], we focused on the effect of NS1619 on PASMCs.

## Conclusion

Our study demonstrates that BK channel opening by NS1619 might be an option for treating pulmonary hypertension. Its inhalation reduces right ventricular work and improves myocardial oxygenation. The topical application of NS1619 attenuates PASMC proliferation in cell culture. As a next step, further research work needs to be conducted for investigating the effect of repetitive NS1619 inhalations on the course of PH disease.

## References

[1] Badesch DB, Champion HC, Sanchez MA, Hoeper MM, Loyd JE, et al (2009) Diagnosis and assessment of pulmonary arterial hypertension. J Am Coll Cardiol 54: S55–S66 S0735-1097(09)01214-5 [pii]; 10.1016/j.jacc.2009.04.011 [doi] 19555859

[2] Barst RJ, Gibbs JS, Ghofrani HA, Hoeper MM, McLaughlin VV, et al (2009) Updated evidence-based treatment algorithm in pulmonary arterial hypertension. J Am Coll Cardiol 54: S78–S84 S0735-1097(09)01223-6 [pii]; 10.1016/j.jacc.2009.04.017 [doi] 19555861 PMC3686287

[3] Vang A, Mazer J, Casserly B, Choudhary G (2010) Activation of endothelial BKCa channels causes pulmonary vasodilation. Vascul Pharmacol 53: 122–129 S1537-1891(10)00088-1 [pii]; 10.1016/j.vph.2010.05.001 [doi] 20470901

[4] Edwards G, Niederste-Hollenberg A, Schneider J, Noack T, Weston AH (1994) Ion channel modulation by NS 1619, the putative BKCa channel opener, in vascular smooth muscle. Br J Pharmacol 113: 1538–1547. 7534190 10.1111/j.1476-5381.1994.tb17171.x PMC1510481

[5] Bonnet S, Archer SL (2007) Potassium channel diversity in the pulmonary arteries and pulmonary veins: implications for regulation of the pulmonary vasculature in health and during pulmonary hypertension. Pharmacol Ther 115: 56–69 S0163-7258(07)00076-9 [pii]; 10.1016/j.pharmthera.2007.03.014 [doi] 17583356

[6] Brayden JE, Nelson MT (1992) Regulation of arterial tone by activation of calcium-dependent potassium channels. Science 256: 532–535. 1373909 10.1126/science.1373909

[7] Blackiston DJ, McLaughlin KA, Levin M (2009) Bioelectric controls of cell proliferation: ion channels, membrane voltage and the cell cycle. Cell Cycle 8: 3519–3528. 9888 [pii]. 10.4161/cc.8.21.9888 PMC2862582 19823012

[8] Revermann M, Barbosa-Sicard E, Dony E, Schermuly RT, Morisseau C, et al (2009) Inhibition of the soluble epoxide hydrolase attenuates monocrotaline-induced pulmonary hypertension in rats. J Hypertens 27: 322–331. 19226702 10.1097/hjh.0b013e32831aedfa PMC2863009

[9] Dahal BK, Cornitescu T, Tretyn A, Pullamsetti SS, Kosanovic D, et al (2010) Role of epidermal growth factor inhibition in experimental pulmonary hypertension. Am J Respir Crit Care Med 181: 158–167 200811-1682OC [pii]; 10.1164/rccm.200811-1682OC [doi] 19850946

[10] Archer SL, Souil E, Dinh-Xuan AT, Schremmer B, Mercier JC, et al (1998) Molecular identification of the role of voltage-gated K+ channels, Kv1.5 and Kv2.1, in hypoxic pulmonary vasoconstriction and control of resting membrane potential in rat pulmonary artery myocytes. J Clin Invest 101: 2319–2330 10.1172/JCI333 [doi] 9616203 PMC508821

[11] Zhao YJ, Wang J, Rubin LJ, Yuan XJ (1998) Roles of K+ and Cl- channels in cAMP-induced pulmonary vasodilation. Exp Lung Res 24: 71–83. 9457470 10.3109/01902149809046055

[12] Barnes PJ, Liu SF (1995) Regulation of pulmonary vascular tone. Pharmacol Rev 47: 87–131. 7784481

[13] Nelson MT, Quayle JM (1995) Physiological roles and properties of potassium channels in arterial smooth muscle. Am J Physiol 268: C799–C822. 7733230 10.1152/ajpcell.1995.268.4.C799

[14] Bonnet S, Savineau JP, Barillot W, Dubuis E, Vandier C, et al.. (2003) Role of Ca(2+)-sensitive K(+) channels in the remission phase of pulmonary hypertension in chronic obstructive pulmonary diseases. Cardiovasc Res 60: 326–336. S0008636303005273 [pii]. 10.1016/s0008-6363(03)00527-3 14613862

[15] Bae YM, Park MK, Lee SH, Ho WK, Earm YE (1999) Contribution of Ca2+-activated K+ channels and non-selective cation channels to membrane potential of pulmonary arterial smooth muscle cells of the rabbit. J Physiol 514 (Pt 3): 747–758. 10.1111/j.1469-7793.1999.747ad.x PMC2269107 9882747

[16] Nagaoka T, Fagan KA, Gebb SA, Morris KG, Suzuki T, et al (2005) Inhaled Rho kinase inhibitors are potent and selective vasodilators in rat pulmonary hypertension. Am J Respir Crit Care Med 171: 494–499 200405-637OC [pii]; 10.1164/rccm.200405-637OC [doi] 15563635

[17] White RE, Kryman JP, El-Mowafy AM, Han G, Carrier GO (2000) cAMP-dependent vasodilators cross-activate the cGMP-dependent protein kinase to stimulate BK(Ca) channel activity in coronary artery smooth muscle cells. Circ Res 86: 897–905. 10785513 10.1161/01.res.86.8.897

[18] Bolotina VM, Najibi S, Palacino JJ, Pagano PJ, Cohen RA (1994) Nitric oxide directly activates calcium-dependent potassium channels in vascular smooth muscle. Nature 368: 850–853 10.1038/368850a0 [doi] 7512692

[19] Barman SA, Zhu S, Han G, White RE (2003) cAMP activates BKCa channels in pulmonary arterial smooth muscle via cGMP-dependent protein kinase. Am J Physiol Lung Cell Mol Physiol 284: L1004–L1011 10.1152/ajplung.00295.2002 [doi];00295.2002 [pii] 12547730

[20] Montani D, Chaumais MC, Guignabert C, Gunther S, Girerd B, et al.. (2013) Targeted therapies in pulmonary arterial hypertension. Pharmacol Ther. S0163-7258(13)00207-6 [pii];10.1016/j.pharmthera.2013.10.002 [doi]. 10.1016/j.pharmthera.2013.10.002 24134901

[21] Carlino C, Tobias JD, Schneider RI, Heller RL, Alpert MA, et al (2010) Pulmonary hemodynamic response to acute combination and monotherapy with sildenafil and brain natriuretic peptide in rats with monocrotaline-induced pulmonary hypertension. Am J Med Sci 339: 55–59 10.1097/MAJ.0b013e3181c078d7 [doi] 19996941

[22] Falcao-Pires I, Goncalves N, Henriques-Coelho T, Moreira-Goncalves D, Roncon-Albuquerque R, et al (2009) Apelin decreases myocardial injury and improves right ventricular function in monocrotaline-induced pulmonary hypertension. Am J Physiol Heart Circ Physiol 296: H2007–H2014 00089.2009 [pii]; 10.1152/ajpheart.00089.2009 [doi] 19346461

[23] Cui B, Cheng YS, Dai DZ, Li N, Zhang TT, et al (2009) CPU0213, a non-selective ETA/ETB receptor antagonist, improves pulmonary arteriolar remodeling of monocrotaline-induced pulmonary hypertension in rats. Clin Exp Pharmacol Physiol 36: 169–175 CEP5044 [pii]; 10.1111/j.1440-1681.2008.05044.x [doi] 18986320

[24] Xu W, Liu Y, Wang S, McDonald T, Van Eyk JE, et al (2002) Cytoprotective role of Ca2+- activated K+ channels in the cardiac inner mitochondrial membrane. Science 298: 1029–1033 10.1126/science.1074360 [doi];298/5595/1029 [pii] 12411707

[25] Dimitropoulou C, White RE, Ownby DR, Catravas JD (2005) Estrogen reduces carbachol-induced constriction of asthmatic airways by stimulating large-conductance voltage and calcium-dependent potassium channels. Am J Respir Cell Mol Biol 32: 239–247 2004-0331OC [pii]; 10.1165/rcmb.2004-0331OC [doi] 15626773

[26] Bowring NE, Arch JR, Buckle DR, Taylor JF (1993) Comparison of the airways relaxant and hypotensive potencies of the potassium channel activators BRL 55834 and levcromakalim (BRL 38227) in vivo in guinea-pigs and rats. Br J Pharmacol 109: 1133–1139. 8401925 10.1111/j.1476-5381.1993.tb13740.x PMC2175747

[27] Short AD, Bian J, Ghosh TK, Waldron RT, Rybak SL, et al (1993) Intracellular Ca2+ pool content is linked to control of cell growth. Proc Natl Acad Sci U S A 90: 4986–4990. 8389460 10.1073/pnas.90.11.4986 PMC46638

[28] Lang F, Foller M, Lang KS, Lang PA, Ritter M, et al (2005) Ion channels in cell proliferation and apoptotic cell death. J Membr Biol 205: 147–157 10.1007/s00232-005-0780-5 [doi] 16362503

[29] Cook SJ, Lockyer PJ (2006) Recent advances in Ca(2+)-dependent Ras regulation and cell proliferation. Cell Calcium 39: 101–112 S0143-4160(05)00206-X [pii]; 10.1016/j.ceca.2005.10.014 [doi] 16343616

[30] Dick GM, Tune JD (2010) Role of potassium channels in coronary vasodilation. Exp Biol Med (Maywood) 235: 10–22 235/1/10 [pii]; 10.1258/ebm.2009.009201 [doi] 20404014

[31] Perros F, Montani D, Dorfmuller P, Durand-Gasselin I, Tcherakian C, et al (2008) Platelet-derived growth factor expression and function in idiopathic pulmonary arterial hypertension. Am J Respir Crit Care Med 178: 81–88 200707-1037OC [pii]; 10.1164/rccm.200707-1037OC [doi] 18420966

[32] Humbert M, Monti G, Fartoukh M, Magnan A, Brenot F, et al (1998) Platelet-derived growth factor expression in primary pulmonary hypertension: comparison of HIV seropositive and HIV seronegative patients. Eur Respir J 11: 554–559. 9596101

[33] Schermuly RT, Dony E, Ghofrani HA, Pullamsetti S, Savai R, et al (2005) Reversal of experimental pulmonary hypertension by PDGF inhibition. J Clin Invest 115: 2811–2821 10.1172/JCI24838 [doi] 16200212 PMC1236676

[34] Ghofrani HA, Seeger W, Grimminger F (2005) Imatinib for the treatment of pulmonary arterial hypertension. N Engl J Med 353: 1412–1413 353/13/1412 [pii]; 10.1056/NEJMc051946 [doi] 16192491

[35] Pullamsetti S, Krick S, Yilmaz H, Ghofrani HA, Schudt C, et al (2005) Inhaled tolafentrine reverses pulmonary vascular remodeling via inhibition of smooth muscle cell migration. Respir Res 6: 128 1465-9921-6-128 [pii]; 10.1186/1465-9921-6-128 [doi] 16262900 PMC1291406

[36] Hoeper MM, Barst RJ, Bourge RC, Feldman J, Frost AE, et al (2013) Imatinib mesylate as add-on therapy for pulmonary arterial hypertension: results of the randomized IMPRES study. Circulation 127: 1128–1138 CIRCULATIONAHA.112.000765 pii]; 10.1161/CIRCULATIONAHA.112.000765 [doi] 23403476

[37] Chang H, Ma YG, Wang YY, Song Z, Li Q, et al (2011) High glucose alters apoptosis and proliferation in HEK293 cells by inhibition of cloned BK Ca channel. J Cell Physiol 226: 1660–1675 10.1002/jcp.22497 [doi] 21413024 10.1002/jcp.22497

[38] Han X, Xi L, Wang H, Huang X, Ma X, et al (2008) The potassium ion channel opener NS1619 inhibits proliferation and induces apoptosis in A2780 ovarian cancer cells. Biochem Biophys Res Commun 375: 205–209 S0006-291X(08)01494-0 [pii]; 10.1016/j.bbrc.2008.07.161 [doi] 18706395

[39] Costa RS, Assreuy J (2005) Multiple potassium channels mediate nitric oxide-induced inhibition of rat vascular smooth muscle cell proliferation. Nitric Oxide 13: 145–151 S1089-8603(05)00076-5 [pii]; 10.1016/j.niox.2005.05.010 [doi] 15993634

[40] Xu W, Liu Y, Wang S, McDonald T, Van Eyk JE, et al (2002) Cytoprotective role of Ca2+- activated K+ channels in the cardiac inner mitochondrial membrane. Science 298: 1029–1033 10.1126/science.1074360 [doi];298/5595/1029 [pii] 12411707

[41] Bentzen BH, Osadchii O, Jespersen T, Hansen RS, Olesen SP, et al (2009) Activation of big conductance Ca(2+)-activated K (+) channels (BK) protects the heart against ischemia-reperfusion injury. Pflugers Arch 457: 979–988 10.1007/s00424-008-0583-5 [doi] 18762970

[42] Redel A, Lange M, Jazbutyte V, Lotz C, Smul TM, et al.. (2008) Activation of mitochondrial large-conductance calcium-activated K+ channels via protein kinase A mediates desflurane-induced preconditioning. Anesth Analg 106: 384–91, table. 106/2/384 [pii];10.1213/ane.0b013e318160650f [doi]. 10.1213/ane.0b013e318160650f 18227289

[43] Yun J, Park H, Ko JH, Lee W, Kim K, et al (2010) Expression of Ca2+ -activated K+ channels in human dermal fibroblasts and their roles in apoptosis. Skin Pharmacol Physiol 23: 91–104 000265680 [pii]; 10.1159/000265680 [doi] 20016251

[44] Krick S, Platoshyn O, McDaniel SS, Rubin LJ, Yuan JX (2001) Augmented K(+) currents and mitochondrial membrane depolarization in pulmonary artery myocyte apoptosis. Am J Physiol Lung Cell Mol Physiol 281: L887–L894. 11557592 10.1152/ajplung.2001.281.4.L887

[45] Aranda M, Bradford KK, Pearl RG (1999) Combined therapy with inhaled nitric oxide and intravenous vasodilators during acute and chronic experimental pulmonary hypertension. Anesth Analg 89: 152–158. 10389795 10.1097/00000539-199907000-00027

[46] Tuder RM, Abman SH, Braun T, Capron F, Stevens T, et al (2009) Development and pathology of pulmonary hypertension. J Am Coll Cardiol 54: S3–S9 S0735-1097(09)01212-1 [pii]; 10.1016/j.jacc.2009.04.009 [doi] 19555856

[47] Sandow SL, Grayson TH (2009) Limits of isolation and culture: intact vascular endothelium and BKCa. Am J Physiol Heart Circ Physiol 297: H1–H7 00042.2009 [pii]; 10.1152/ajpheart.00042.2009 [doi] 19411289

